# Association between the expression of lncRNA BASP-AS1 and volume of right hippocampal tail moderated by episode duration in major depressive disorder: a CAN-BIND 1 report

**DOI:** 10.1038/s41398-021-01592-4

**Published:** 2021-09-08

**Authors:** Antoine Yrondi, Laura M. Fiori, Nikita Nogovitsyn, Stefanie Hassel, Jean François Théroux, Zahia Aouabed, Benicio N. Frey, Raymond W. Lam, Roumen Milev, Daniel J. Müller, Jane A. Foster, Claudio Soares, Susan Rotzinger, Stephen C. Strother, Glenda M. MacQueen, Stephen R. Arnott, Andrew D. Davis, Mojdeh Zamyadi, Jacqueline Harris, Sidney H. Kennedy, Gustavo Turecki

**Affiliations:** 1 McGill Group for Suicide Studies, Douglas Mental Health University Institute, Department of Psychiatry, McGill University, Montreal, QC Canada; 2 Department of Psychiatry, Cumming School of Medicine, University of Calgary, Calgary, AB Canada; 3 Mathison Centre for Mental Health Research and Education, University of Calgary, Calgary, AB Canada; 4 Hotchkiss Brain Institute, University of Calgary, Calgary, AB Canada; 5 Department of Psychiatry & Behavioural Neurosciences, McMaster University and St Joseph’s Healthcare Hamilton, Hamilton, ON Canada; 6 Department of Psychiatry, University of British Columbia, Vancouver, BC Canada; 7 Department of Psychiatry at Queens Providence Care Hospital, Kingston, ON Canada; 8 Department of Psychiatry, University Health Network, Krembil Research Institute, University of Toronto, Toronto, ON Canada; 9 Centre for Addiction and Mental Health, Toronto, ON Canada; 10 St Michael’s Hospital, Li Ka Shing Knowledge Institute, Centre for Depression and Suicide Studies, Toronto, ON Canada; 11 Department of Psychiatry, Queen’s University, Kingston, ON Canada; 12 Rotman Research Institute, Baycrest Health Sciences; Department of Medical Biophysics, University of Toronto, Toronto, ON Canada; 13 Department of Computer Science, University of Alberta, Edmonton, AB Canada

**Keywords:** Depression, Epigenetics and behaviour, Molecular neuroscience

## Abstract

The pathophysiology of major depressive disorder (MDD) encompasses an array of changes at molecular and neurobiological levels. As chronic stress promotes neurotoxicity there are alterations in the expression of genes and gene-regulatory molecules. The hippocampus is particularly sensitive to the effects of stress and its posterior volumes can deliver clinically valuable information about the outcomes of antidepressant treatment. In the present work, we analyzed individuals with MDD (*N* = 201) and healthy controls (HC = 104), as part of the CAN-BIND-1 study. We used magnetic resonance imaging (MRI) to measure hippocampal volumes, evaluated gene expression with RNA sequencing, and assessed DNA methylation with the (Infinium MethylationEpic Beadchip), in order to investigate the association between hippocampal volume and both RNA expression and DNA methylation. We identified 60 RNAs which were differentially expressed between groups. Of these, 21 displayed differential methylation, and seven displayed a correlation between methylation and expression. We found a negative association between expression of Brain Abundant Membrane Attached Signal Protein 1 antisense 1 RNA (BASP1-AS1) and right hippocampal tail volume in the MDD group (*β* = −0.218, *p* = 0.021). There was a moderating effect of the duration of the current episode on the association between the expression of BASP1-AS1 and right hippocampal tail volume in the MDD group (*β* = −0.48, 95% C.I. [−0.80, −0.16]. *t* = −2.95 *p* = 0.004). In conclusion, we found that overexpression of BASP1-AS1 was correlated with DNA methylation, and was negatively associated with right tail hippocampal volume in MDD.

## Introduction

Major depressive disorder (MDD) is one of the leading causes of morbidity and disability worldwide. With more than 300 million people affected, and a lifetime prevalence of between 16 and 17% in the general population [1], MDD is an increasingly widespread illness [2]. Currently, there are no reliable biological tests or biomarkers to diagnose MDD. Moreover, the pathophysiological mechanisms underlying MDD are poorly understood. However, neuroimaging and biological studies may provide insight into the pathophysiology of depression and potentially aid in the diagnostic process.

Stressful life events can play a role in triggering the onset of an initial Major Depressive Episode (MDE); however, their role in episode onset seems to progressively diminish as the number of episodes increases [3, 4]. Biological changes that mediate the interplay between external stressors and recurrence could be involved in illness progression. One candidate mechanism is structural abnormalities within the hippocampus. This region is known to regulate behavioral and neuroendocrine responses to stress and can be sensitive to excessive exposure to stress-induced release of steroidal and inflammatory signaling molecules [5, 6]. Indeed, the hippocampus seems to be a highly stress-sensitive brain region [7, 8] and MDD is a highly stress-sensitive illness [9]. Moreover, preclinical studies suggest that stress can result in structural changes to the hippocampus [10, 11]. Also, several metanalyses of magnetic resonance imaging (MRI) data suggest that a reduction in hippocampal volume is associated with MDD [12–14], with moderate effect sizes (Cohen’s d range = 2.41–2.47) [14–16]. Most of these studies focused on the whole hippocampus, while only a few have investigated hippocampal subfields [17, 18]. In particular, Malykhin et al. highlighted a reduction of the hippocampal tail during an MDE [18]. In a recent CAN-BIND report, Nogovitsyn et al. replicated the results published by Maller et al. [19] in a large independent cohort, supporting the notion that pre-treatment hippocampal tail (Ht) volumetry may have the capacity to predict clinical outcomes of antidepressant treatment in patients with MDD [20]. Studies have also shown that hippocampal volume reduction is associated with both the duration of the current episode and the number of previous episodes [21–24]. However, one metanalysis indicated that the hippocampal volume reduction was found only for episodes of greater than 2 years duration, and for recurrent MDD [25]. Chronic stress appears to play an important role in the pathological decrease of hippocampal volume through neurotoxic processes, involving hypothalamic-pituitary-adrenal (HPA) axis dysregulation, inflammation, oxidative stress and altered neurotrophic signaling [7, 26, 27].

Several studies have suggested that risk genotypes of candidate genes or psychological stress do not directly modulate clinical symptoms, but regulate brain structure/function through cellular and molecular mechanisms [28]. In animal models, a history of stress exposure can permanently alter both gene expression patterns in the hippocampus and behavioral response to a novel stressor [29]. Moreover, chronic stress may induce various epigenetic changes in multiple signaling pathways, including the HPA axis, brain-derived neurotrophic factor (BDNF) signaling [30], and in different neural structures, such as the hippocampus, with a subsequent effect on their functions [31]. For example, early-life stress is associated with both hypomethylated and hypermethylated promoters in human hippocampal tissue, suggesting that active DNA methylation and demethylation may result from environmental stressors [32]. Labonté et al. suggested that differential methylation associated with early life stress occurs across a number of biological processes [32]. Moreover, epigenetic mechanisms have been involved in the regulation of adult neurogenesis in animal models [33]. Therefore, chronic stress may promote neurotoxicity by activating molecular cascades, which in turn are regulated by molecules such as non-coding RNA (ncRNA) and other RNA species [34, 35]. These molecular cascades could be associated directly or indirectly with brain alterations, mainly in the hippocampus. Therefore, in the current study, we aimed to assess the association between hippocampal tail (Ht) volume and RNA expression regulated by epigenetic mechanisms, in a cohort of MDD and healthy participants, who were part of the Canadian Biomarker Integration Network in Depression (CAN-BIND)-1 study.

## Methods and materials

### Participants

Participants completed a two-phase clinical observational study designed to evaluate biomarkers associated with response to a selective serotonin reuptake inhibitor (SSRI) and subsequently, for non-responders to the SSRI (escitalopram), to a serotonin-dopamine modulator (aripiprazole) [36]. The MDD sample was between the ages of 18 and 60 years, with a Montgomery-Asberg Depression Rating Scale (MADRS) [37] score of 24 or greater, and also consisted of sex- and age-matched healthy controls (HC, *n* = 104) (see Table 1). Participants were recruited at six academic centers across Canada between August 2013 and December 2016 [36]. The Mini-International Neuropsychiatric Interview (MINI) [38] Version 6.1 was administered to confirm or rule-out MDD status and the presence or absence of other psychiatric comorbidities. Exclusion criteria included a diagnosis of bipolar disorder, and high suicide risk and/or psychosis in the current MDE. Failure to respond after four or more adequate antidepressant trials in the current episode or previous failure to respond to escitalopram or aripiprazole were also exclusion criteria. Importantly, all MDD participants were free from any psychotropic medications and were required to undergo a wash out period of at least five half-lives before entering the study. For a detailed description of inclusion and exclusion criteria, see Table 1 in Lam et al. [39]. All participants provided written informed consent, and ethics approval was obtained at each center. The trial was registered at ClinicalTrials.gov (identifier: NCT01655706).

**Table 1:** Socio-demographic characteristics of study participants.

	MDD (*N* = 201)	HC (*N* = 104)	*p* value
Age (SD)	35.26 (12.62)	33.10 (10.73)	0.12^a^
Sex (%Female)	125 (62.2%)	66 (63.5%)	0.83^b^
MADRS (SD)	29.89 (5.46)	0.85 (1.72)	<0.001^a^
Ethnicity	146 (69.5%)	71 (68.2%)	0.43^b^
Caucasian	32 (15.9%)	23 (22.1%)	0.18^b^
Asian	13 (6.5%)	3 (2.9%)	0.18^b^
Hispanic	10 (5%)	2 (1.9%)	0.19^b^
Black	16 (8%)	4 (3.8%)	0.16^b^
Others	−	2	
No answer			
Duration of current episode-months (SD)	25.79 (33.61)	−	

*HC* healthy control, *MADRS* Montgomery Asberg Depression Rating Scale, *MDD* Major Depressive Disorder, *N* = number, *SD* standard deviation.

^a^*t* test.

^b^Chi-2.

### MRI data acquisition and processing

All details of the CAN-BIND neuroimaging acquisition protocols and procedures for data quality control were published elsewhere [40], but briefly, all sites followed harmonized MRI acquisition protocols performed on 3 T MR scanners located at six different academic institutions. A whole-brain T_1_- weighted turbo gradient echo sequence was acquired at 1 mm^3^ resolution. Structural brain images were acquired with the following parameters: repetition time (TR) = 6.4–1900 ms; echo time (TE) = 2.2–3.4 ms; flip 8–15°; inversion time (TI) = 450–950 ms; field of view (FOV) 256 mm; matrix dimensions 220 × 220 and 256 × 256; contiguous slices at 1 mm thickness.

Hippocampal volume measurements were obtained using a subfield-specific segmentation workflow [41] that was integrated as a part of FreeSurfer version 6.0 (http://surfer.nmr.mgh.harvard.edu/). This segmentation workflow generated bilateral total hippocampal volume (THV) as well as 12 additional segmentations for hippocampal subregions including hippocampal tail (Ht), subiculum, fissure, presubiculum, parasubiculum, molecular layer (ML), granule cell layer and molecular layer of the dentate gyrus (GC-ML-DG), fimbria, the cornu ammonis (CA) area subdivided into CA1, CA2/3, CA4, and hippocampal amygdala transition area (HATA). For the present analysis, we used neuroimaging data that were generated for a previous CAN-BIND-1 report, which also included a three-step quality control procedure for the hippocampal segmentations [20]. To reduce site scanner effects, following MacQueen et al. (2019), the imaging group made efforts at all phases of data acquisition and management to maintain consistent neuroimaging protocols, centralized data collection and quality assurance. Human and lego phantoms were used and we have reported on this methodology and issues with site variability previously [40]. Second, the FreeSurfer version 6.0 segmentation data employed in the present work relied on the probabilistic algorithm [41] that has already been assessed for the test-retest/scan-rescan reliability considering existing differences between scanner parameters [42]. Third, to control for possible between-site variance in hippocampal volumes we used a backward multiple regression model as described in Nogovistyn et al. 2020 [20].

### Biological assessments

#### RNA extraction and sequencing

Whole blood for RNA analysis was collected at baseline and filtered using LeukoLOCK filters (Life Technologies). Total RNA was extracted using a modified version of the LeukoLOCK Total RNA Isolation System protocol, which included DNase treatment to remove genomic DNA. The quality of RNA was assessed using the Agilent 2200 Tapestation, and only samples with RNA Integrity Number (RIN) ≥ 6.0 were used. All libraries were prepared using the Illumina TruSeq mRNA stranded protocol following the manufacturer’s instructions. Samples were sequenced at the McGill University and Genome Quebec Innovation Centre (Montreal, Canada) using the Illumina HiSeq4000 with 100nt paired-end reads. FASTXToolkit [43] and Trimmomatic [44] were respectively used for quality and adapter trimming. Tophat2, using bowtie2 was used to align the cleaned reads to reference genome (GRCh38). Reads that lost their pairs through the cleaning process were aligned independently from the reads that still had pairs. Quantification on each gene’s expression was estimated using HTSeq-count and a reference transcript annotation from ENSEMBL. Counts for the paired and orphaned reads for each sample were added to each-other. Normalization was conducted on the resulting gene matrix using DESeq2.

### Genome-wide DNA methylation analysis on the Infinium MethylationEPIC Beadchip

DNA was extracted from whole blood samples obtained from healthy controls and MDD participants at baseline, using a modified version of the Qiagen FlexiGene DNA kit as described in Ju et al. [45]. Bisulfite conversion, DNA quality control, genome-wide methylation analysis, and initial methylation signal detection quality control was performed at the McGill University and Genome Quebec Innovation Center (GQ). The Infinium MethylationEPIC Beadchip was used to assess genome-wide DNA methylation (Illumina, US). After accounting for attrition rates, and DNA sample quality control, pre-processing and analysis of raw microarray data for the remaining samples was conducted within R (version 3.4) predominantly using the Chip Analysis Methylation Pipeline (ChAMP) Bioconductor package [46], which utilizes many elements of minfi [47]. Sample methylation signal QC was assessed by plotting log median methylated and unmethylated signals. Samples were removed if they failed to cluster with others or if they exhibited lower median intensities in either signal channel. Probes with low signal detection relative to control probes, probes with <3 beads in >5% of samples, cross reactive probes, non-CpG probes, sex chromosome probes, and probes that hybridize to single nucleotide polymorphism sites were removed. Beta (β) values were calculated as the ratio of methylated signal to the sum of unmethylated and methylated signals at each CpG site, and subsequently normalized. Log2 transformed β values were used for the remainder of pre-processing steps as recommended by Du et al. [48], but reported as β values. Technical batches and covariates were detected using single value decomposition analysis. Detected and known batch effects were corrected for prior to differential methylation analysis. The CpG site annotations are based on the chip manifest (the manifest uses information from the UCSC). CpG sites are annotated to a gene if they are in the body or less than 1500 bp upstream of the transcription start site (TSS). Technical batches and covariates were detected using single value decomposition analysis. Detected and known batch effects were corrected for prior to differential methylation analysis.

### Investigating effects of blood cell heterogeneity

Heterogeneity of white blood cell types has potential confounding effects on DNA methylation measurements based in peripheral blood samples [49]. To address the possibility of confounding effects of blood cell composition, complete blood cell counts were obtained from each patient during the trial. Linear regressions were used to assess effects of cell composition on DNA methylation.

### Statistical analysis

Sociodemographic and clinical characteristics are presented using means and standard deviations for continuous variables and frequency distributions for categorical variables. As part of data preparation, all RNA expression values were log2 transformed. The following covariates: sex, age and RIN were used in the general linear model (GLM) implementation of DESeq2 to perform the differential analysis. We focused on RNAs with fold changes greater than 20% between MDD and HC. We used a False Discovery Rate (FDR) threshold of 5% for each multiple comparison. For selected genes, we compared the level of methylation (β values) for each CpG site annotated to that gene using t-tests. We used a FDR threshold of 5% to identify significant CpG sites within each gene region. For each CpG site with significant methylation differences between cases and controls, we assessed the association between these β values and the expression of the corresponding RNA using linear regressions. To assess the association between the expression of selected RNAs and volume of the different substructures of the hippocampus, we used linear regressions. We used a FDR threshold of 5% to correct for multiple testing. We performed separate identical designs for each hippocampus subfield. For RNAs whose expression was significantly associated with volume of one of substructures of the hippocampus, we included clinical features (MADRS score and duration of current episode) and total brain volume as covariates in our model. Moreover, we assessed the moderating effect of the duration of the current episode, using Hayes’ model (model 1) with age and sex as co-variables [50]. Moderation analysis is used to address, when, or under what circumstances that effect exists or does not and in what magnitude. We would like to show that RNA’s effect on hippocampal tail volume depends in some way on the duration of the current episode. More specifically, RNA’s effect on hippocampal tail volume will be said to be moderated by the duration of the current episode if the size of RNA’s effect on hippocampal tail volume varies with the duration of the current episode [51].

Statistical analyses were performed with SPSS 25.0 (IBM Corp. Released 2017. IBM SPSS Statistics for Mac, Version 25.0. Armonk, NY: IBM Corp.).

## Results

### Demographic data

Data were collected for 211 depressed and 112 healthy participants. Samples from 10 MDD and 8 HC participants were excluded due to poor RNA quality, leaving a total of 201 MDD and 104 HC samples for analyses. There was no difference between groups for age or sex (Table 1). When we integrated baseline structural MRI and molecular data, combined data were available from 188 depressed and 103 healthy participants who did not differ from the larger group in terms of clinical or socio-demographic variables.

### RNA expression and DNA methylation

We identified 60 RNAs which were differentially expressed between groups. Among these, we identified 21 genes that were differentially methylated between groups (Table S1). Seven of these genes displayed both differential methylation between MDD and HC groups and a significant correlation between methylation and expression (Table 2). Expression of two of these genes, Brain Abundant Membrane Attached Signal Protein 1 antisense 1 RNA (BASP1-AS1) and Interleukin 18 Receptor Accessory Protein (IL18RAP), were each associated with three differentially methylated sites, whereas the expression of the remaining five genes [ArfGAP With GTPase Domain, Ankyrin Repeat And PH Domain 1 (AGAP1), Alpha Kinase 1 (ALPK1), Phosphatidylinositol-4,5-Bisphosphate 3-Kinase Catalytic Subunit Delta antisense RNA1 (C1orf200), Membrane Bound O-Acyltransferase Domain Containing 2 (MBOAT2) and Long Intergenic Non-Protein Coding RNA 1270 (LINC01270)] were each associated with one differentially methylated site. BASP1-AS1, C1orf200 and LINC01270 are long non-coding RNAs (lncRNA). We found no effects of blood cell composition on DNA methylation (Table S2).

**Table 2:** Differential methylation between MDD and HC groups for genes displaying differential expression and an association between RNA expression and methylation.

RNA	CpG site	Delta between MDD and HC (%)	*p* value	*β*	*p* value
AGAP1	cg11553308	0.8	0.049	−0.231	<0.001
ALPK1	cg15936366	0.4	0.04	0.192	0.001
BASP1-AS1	cg20475607	0.5	0.034	0.178	0.006
	cg07234569	−0.3	0.034	−0.170	0.009
	cg25203704	−0.4	0.034	−0.154	0.024
C1orf200	cg16597045	−0.7	0.045	−0.280	<0.001
IL18RAP	cg23256822	1.4	0.016	0.48	<0.001
	cg03938978	8.5	0.012	0.581	<0.001
	cg12278959	2.9	0.018	0.49	<0.001
MBOAT2	cg27441011	0.3	0.034	0.153	0.009
LINCO1270	cg02110603	0.7	0.024	0.262	<0.001

*CpG* cytosine-guanine, *HC* healthy control, *MDD* Major Depressive Disorder.

### Association between RNA expression and volume of substructures of the hippocampus

**Fig. 1:**
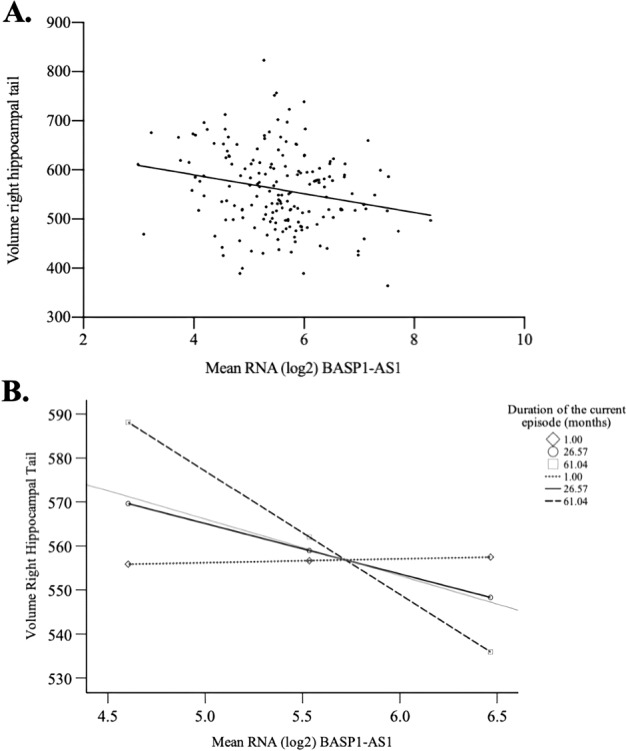
Association between the right hippocampal tail and BASP1-AS1. **A** Association between volume of the right hippocampal tail and BASP1-AS1 in the MDD group; **B** Influence of duration of current episode on the association between volume of the right hippocampal tail and BASP1-AS1 in the MDD group.

With regards to right hippocampal tail volume, we found a negative association between expression of BASP1-AS1 and right hippocampal tail volume in the MDD group (*β* = −0.218, *p* = 0.021) (Fig. 1A). There was a trend for a negative association between right hippocampal tail volume and expression of (i) MBOAT2 (*β* = −0.171, *p* = 0.051) and (ii) ALPK1 (*β* = −0.176, *p* = 0.051) (Table 3). We did not find any association between the seven identified RNAs and the right hippocampal tail volume in the healthy control group (Table 3). Moreover, the association between expression of BASP1-AS1 and right hippocampal tail volume in the MDD group remained significant when we added clinical features (MADRS and duration of current episode), total brain volume, age and sex as covariates (Table 4). A moderation analysis with age and sex as co-variables suggested a significant moderating effect of the duration of the current episode of MDD on the association between the expression of BASP1-AS1 and right hippocampal tail volume in the MDD group (*β* = −0.48, 95% C.I. [−0.80, −0.16]. *t* = −2.95 *p* = 0.004). We highlighted that the duration of current episode could influence the association between volume of the right hippocampal tail and BASP1-AS1 in the MDD group. Indeed, the expression of BASP1-AS1 was only influential in reducing right hippocampal tail volume (Fig. 1B) when the current episode was longer than 28 months.

**Table 3:** Association between RNA expression and right hippocampal tail volumes in MDD and HC groups.

RNA	MDD		HC	
	*β*	p value	*β*	p value
BASP1-AS1	−0.218	0.021	−0.032	0.999
MBOAT2	−0.171	0.051	0.021	0.999
ALPK1	−0.176	0.051	0.044	0.999
IL18RAP	−0.09	0.266	−0.058	0.999
LINCO1270	−0.057	0.45	−0.087	0.999
C1orf200	−0.16	0.057	0.131	0.999
AGAP1	0.102	0.244	−0.011	0.999

*HC* healthy control, *MDD* Major Depressive Disorder.

**Table 4:** Association between BASP1-AS1 expression and right hippocampal tail volumes in MDD.

Predictor	*β*	*p* value
BASP-AS1	−0.143	0.027
AGE	−0.019	0.791
Total brain volume	0.611	<0.001
MADRS	−0.061	0.342
Duration current MDE	−0.016	0.807
SEX:		
M – F	−0.187	0.304

*F* Female, *M* Male, *MADRS* Montgomery Asberg Depression Rating Scale, *MDE* Major Depressive Episode.

In addition to this, we also examined whether the reported association is exclusive to the right tail of the hippocampus, or if extends to other subfields of the hippocampus. We found no association between the seven identified RNAs and left hippocampal tail volume in either the MDD nor the healthy control groups (Tables S3, S4). In addition, we did not detect any significant associations between the RNAs and the rest of the subfields of the hippocampus (for details see Table S5).

## Discussion

In the present study, we investigated associations between the volumes of hippocampus and RNA expression in patients with MDD. We showed a negative association between the right hippocampal tail volume and BASP1-AS1 expression, which was moderated by the duration of the current episode of MDD. BASP1-AS1 is an antisense lncRNA. LncRNAs are defined as RNA molecules greater than 200 nucleotides in length with low protein-coding potential. They are found throughout the genome and are generally categorized based on their relation to other known genes. lncRNAs are annotated on the basis of their genomic position with respect to the protein-coding genes. According to this classification, lncRNA biotypes broadly fall into two types—genic lncRNA (sense or antisense) and intergenic [52, 53]. LncRNAs may interact with DNA, RNA or protein molecules [54]. They are engaged in diverse structural, functional, and regulatory activities, and have roles in nuclear organization and transcriptional, post-transcriptional, and epigenetic processes [55]. LncRNAs derived from antisense transcription are implicated in the regulation of sense protein-coding genes. Indeed, antisense transcription has been ascribed roles in gene regulation involving degradation of the corresponding sense transcripts (RNA interference). It has also been involved in gene silencing at the chromatin level [56]. Although the exact physiological role of BASP1-AS1 in the brain is not fully understood, it could regulate the expression of BASP1. Indeed, a recent study, Prajapati et al. highlighted, in “in vitro” model, that BASP1- AS1 regulates BASP1 in human neural progenitor cells, and has a critical role in neuronal differentiation [57]. It seemed that over-expression of BASP1 in adult neurons promotes sprouting [58] and, more specifically, modulate neurite outgrowth in hippocampal neurons [59]. Moreover, BASP1, is highly expressed in neurons during brain development [59]. In this study, we showed that BASP1-AS1 expression was higher in patients with MDD compared to healthy controls. The negative association between the right hippocampal tail volume and BASP1-AS1 is strongest for episodes that have progressed for more than two years. This negative association could be explained by a negative regulation of expression of BASP1, involved in neurite growth and in neural differentiation. This association could be a biomarker of the duration of the current episode. This finding is of importance to clinical practice because it is known that longer duration of a current episode is associated with non-response and resistance to the treatment [60, 61]. This finding could represent potential treatment targets in future. These data are also consistent with literature highlighting associations between hippocampal plasticity and growth factors [62]. The fact that our associations were only found with the right hippocampal tail is consistent with previous reports that reduction in hippocampal volume is greater in the right hemisphere [13, 63].

Although blood samples allow simultaneous investigation of DNA methylation and RNA expression, blood analyses have significant limitations. The relationship between epigenomic and proteomic peripheral changes measured in the periphery and those in the central nervous system may not always be relevant [64]. However, considering that MDD is a systemic illness, blood samples could provide meaningful insight into underlying mechanisms related to the pathophysiology of this disorder. Moreover, the unbalanced sample size could be considered as a limitation. However, these analyses were from baseline of the CAN-BIND-1 study [39]. The rationale to recruit a greater number of patients at baseline was in the fact that at weeks 8 and 16 of the trial, it was expected that the patients would be distributed into several groups depending on treatment outcomes (ie., early responders, late responders, non-responders). Therefore, in the present analysis, the unbalanced sample size is the inherent ramification of a real-world study.

In the present work, we chose to focus on the hippocampal tail because the extant literature has consistently demonstrated that volumetric measures of the posterior hippocampus are strongly associated with the likelihood of clinical improvements following antidepressant treatment [19, 20, 65]. We would like to acknowledge that the results of the present analysis cannot exclude the possibility that the levels of RNA expression (BASP1-AS1) can also be associated with other stress-sensitive brain regions, including the amygdala, hypothalamus or prefrontal cortex.

We found that overexpression of BASP1-AS1 was correlated with DNA methylation, and was negatively associated with right tail hippocampal volume in MDD. This association was moderated by the duration of the current major depressive episode. As well as representing potential diagnostic biomarkers, RNA and methylation sites associated with neuroimaging represent potential treatment targets, as well as possible biomarkers of treatment response. Although these results are promising, they need to be independently replicated and mechanistic follow up studies should be conducted to confirm these hypotheses.

## Supplementary information

Supplemental Material

Supplemental Table S5
