# Supplementary material for: Association between the expression of lncRNA BASP-AS1 and volume of right hippocampal tail moderated by episode duration in major depressive disorder: a CAN-BIND 1 report
Source: Transl Psychiatry. 2021 Sep 8;11:469. doi: 10.1038/s41398-021-01592-4 (PMC8433329; doi:10.1038/s41398-021-01592-4)
Supplement: Supplementary file 1 — Supplemental Material [file 41398_2021_1592_MOESM1_ESM.docx]

**Supplementary data**

**Table S1**. *RNA expression differences between MDD and healthy controls*

| Gene | baseMean | log2FoldChange | lfcSE | stat | pvalue | FDR |
| --- | --- | --- | --- | --- | --- | --- |
| FOLR3 | 1361.39 | 0.845264072 | 0.15707035 | 5.38143616 | 7.39E-08 | 0.00022153 |
| **NRN1** | 53.3519 | 0.700149959 | 0.1875044 | 3.73404541 | 0.00018843 | 0.04746794 |
| AC092299.7 | 34.26 | 0.581191138 | 0.11505235 | 5.0515366 | 4.38E-07 | 0.00109494 |
| **ZDHHC19** | 49.22 | 0.49689134 | 0.11369095 | 4.37054423 | 1.24E-05 | 0.01548183 |
| FCGR1C | 77.94 | 0.465604438 | 0.12423903 | 3.74765039 | 0.0001785 | 0.04746794 |
| FCGR1A | 1623.28 | 0.445938945 | 0.11858652 | 3.76045217 | 0.00016961 | 0.04746794 |
| **LINC01270** | 370.45 | 0.426317483 | 0.10476965 | 4.0690933 | 4.72E-05 | 0.03075977 |
| **BASP1-AS1** | 51.60 | 0.425291977 | 0.10782125 | 3.94441714 | 8.00E-05 | 0.04143074 |
| RP11-556I13.2 | 170.85 | 0.394952118 | 0.10489339 | 3.76527167 | 0.00016637 | 0.04746794 |
| AC092620.3 | 33.08 | 0.383612664 | 0.08698384 | 4.41016026 | 1.03E-05 | 0.01548183 |
| RP11-6I2.3 | 32.16 | 0.382948665 | 0.10427534 | 3.67247588 | 0.00024021 | 0.04900322 |
| HNRNPA1P52 | 47.33 | 0.3795192 | 0.09841522 | 3.85630586 | 0.00011511 | 0.04746794 |
| CFAP45 | 313.50 | 0.376112673 | 0.08600285 | 4.37325827 | 1.22E-05 | 0.01548183 |
| ALPL | 14973.63 | 0.372538491 | 0.10036428 | 3.71186337 | 0.00020574 | 0.04807668 |
| ADM | 3638.73 | 0.360146766 | 0.09372912 | 3.84242122 | 0.00012183 | 0.04746794 |
| UPK3B | 51.55 | 0.359456786 | 0.09491251 | 3.78724368 | 0.00015233 | 0.04746794 |
| KCNJ2-AS1 | 257.11 | 0.356896979 | 0.07937879 | 4.49612523 | 6.92E-06 | 0.01481933 |
| **RP11-670E13.6** | 230.31 | 0.354891957 | 0.09618603 | 3.68964153 | 0.00022457 | 0.04900322 |
| CSF2RBP1 | 109.98 | 0.33518484 | 0.08856945 | 3.78442947 | 0.00015406 | 0.04746794 |
| AP006222.2 | 161.08 | 0.332112177 | 0.09089205 | 3.65391869 | 0.00025827 | 0.04900322 |
| CTD-3088G3.8 | 2869.18 | 0.325196903 | 0.07980484 | 4.0749021 | 4.60E-05 | 0.03075977 |
| C9orf106 | 40.54 | 0.310947498 | 0.08248707 | 3.76965156 | 0.00016348 | 0.04746794 |
| KCNJ2 | 5603.06 | 0.308531579 | 0.08319526 | 3.70852359 | 0.00020847 | 0.04807668 |
| **ICAM5** | 48.99 | 0.304748929 | 0.07749227 | 3.93263651 | 8.40E-05 | 0.04143074 |
| **IL18RAP** | 4422.15 | 0.29971969 | 0.07988241 | 3.75201131 | 0.00017542 | 0.04746794 |
| **SLC25A37** | 40739.67 | 0.296282387 | 0.06747848 | 4.39076871 | 1.13E-05 | 0.01548183 |
| P4HA2 | 36.99 | 0.295378121 | 0.08170552 | 3.61515517 | 0.00030017 | 0.04999461 |
| PLIN4 | 874.26 | 0.292413121 | 0.080306 | 3.64123624 | 0.00027133 | 0.04900322 |
| **PLIN5** | 932.30 | 0.291670788 | 0.07615096 | 3.83016575 | 0.00012806 | 0.04746794 |
| **AC116366.6** | 155.12 | 0.287320316 | 0.07872869 | 3.64949972 | 0.00026275 | 0.04900322 |
| MYBPC3 | 505.27 | 0.282353707 | 0.0637232 | 4.43094068 | 9.38E-06 | 0.01548183 |
| EMR2 | 9612.81 | 0.276159856 | 0.07292538 | 3.78688245 | 0.00015255 | 0.04746794 |
| **GRB10** | 376.30 | 0.269554311 | 0.07442993 | 3.62158479 | 0.0002928 | 0.04975781 |
| **MBOAT2** | 2332.84 | 0.266151934 | 0.06401402 | 4.15771308 | 3.21E-05 | 0.02840843 |
| **LIMK2** | 10580.63 | 0.257367161 | 0.06893755 | 3.73333785 | 0.00018896 | 0.04746794 |
| AC004069.2 | 124.67 | 0.250360329 | 0.06663812 | 3.75701352 | 0.00017195 | 0.04746794 |
| CTC-251I16.1 | 1955.46 | 0.234252221 | 0.06201838 | 3.7771418 | 0.00015864 | 0.04746794 |
| LINC01503 | 165.32 | 0.229525635 | 0.06242461 | 3.67684523 | 0.00023614 | 0.04900322 |
| **ALPK1** | 4864.10 | 0.228814097 | 0.06223417 | 3.67666329 | 0.0002363 | 0.04900322 |
| NCF4 | 15777.98 | 0.225881089 | 0.05950428 | 3.79604773 | 0.00014702 | 0.04746794 |
| **FAM151B** | 108.34 | 0.224210826 | 0.05864143 | 3.82342001 | 0.00013161 | 0.04746794 |
| **CCDC17** | 222.58 | 0.217536715 | 0.05888645 | 3.69417296 | 0.0002206 | 0.04900322 |
| **PIK3CD-AS1** | 86.87 | 0.212765629 | 0.05866896 | 3.62654491 | 0.00028724 | 0.04975781 |
| NFATC2 | 2073.73 | -0.224335939 | 0.06090485 | -3.6833837 | 0.00023016 | 0.04900322 |
| ZNF831 | 1158.16 | -0.23589797 | 0.06266568 | -3.7643886 | 0.00016696 | 0.04746794 |
| PTCH1 | 583.83 | -0.265487822 | 0.07208874 | -3.6827918 | 0.00023069 | 0.04900322 |
| TRBV2 | 161.61 | -0.282336068 | 0.07164861 | -3.940566 | 8.13E-05 | 0.04143074 |
| CELSR2 | 124.22 | -0.305094037 | 0.08429694 | -3.6192776 | 0.00029543 | 0.04975781 |
| **LGR6** | 271.79 | -0.307916974 | 0.08217472 | -3.7471009 | 0.00017889 | 0.04746794 |
| NCAPG2 | 450.57 | -0.320187776 | 0.08072524 | -3.9663899 | 7.30E-05 | 0.04051157 |
| **AGAP1** | 224.15 | -0.340466098 | 0.09310141 | -3.656938 | 0.00025525 | 0.04900322 |
| **FCGBP** | 459.31 | -0.397314383 | 0.0964378 | -4.1199031 | 3.79E-05 | 0.02840843 |
| NPIPA7 | 47.75 | -0.399492885 | 0.10218384 | -3.9095505 | 9.25E-05 | 0.04143074 |
| IGKV1-6 | 34.55 | -0.407647505 | 0.11220365 | -3.6331039 | 0.00028003 | 0.04975781 |
| COL7A1 | 110.68 | -0.520870344 | 0.13297608 | -3.9170229 | 8.96E-05 | 0.04143074 |
| IGLV3-19 | 163.94 | -0.563404663 | 0.1363207 | -4.1329355 | 3.58E-05 | 0.02840843 |
| **KIF24** | 27.59 | -0.820914073 | 0.14900327 | -5.5093694 | 3.60E-08 | 0.00013496 |
| EEF1A1P13 | 156.25 | -0.91676989 | 0.13951886 | -6.5709386 | 5.00E-11 | 3.75E-07 |
| OTOF | 75.34 | -1.367932896 | 0.23668298 | -5.7795998 | 7.49E-09 | 3.74E-05 |
| AC016739.2 | 26.43 | -2.120846334 | 0.29329635 | -7.2310697 | 4.79E-13 | 7.18E-09 |

***Bold****: gene differentially methylated between groups*

**Table S2.** *Association between blood cell counts and methylation*

| CpG site | Cell Blood | Beta | p-value (FDR) |
| --- | --- | --- | --- |
| cg11553308 | Basophils | -0.035 | 0.47 |
|  | Eosinophils | 0.003 | 0.672 |
|  | Lymphocytes | -0.002 | 0.521 |
|  | Monocytes | 0.009 | 0.47 |
|  | Neutrophils | <0.001 | 0.47 |
| cg15936366 | Basophils | -0.268 | 0.705 |
|  | Eosinophils | -0.021 | 0.745 |
|  | Lymphocytes | -0.02 | 0.705 |
|  | Monocytes | -0.039 | 0.705 |
|  | Neutrophils | -0.012 | 0.705 |
| cg20475607 | Basophils | -0.304 | 0.786 |
|  | Eosinophils | -0.023 | 0.788 |
|  | Lymphocytes | -0.025 | 0.786 |
|  | Monocytes | -0.044 | 0.786 |
|  | Neutrophils | -0.016 | 0.786 |
| cg07234569 | Basophils | -0.366 | 0.699 |
|  | Eosinophils | -0.039 | 0.703 |
|  | Lymphocytes | -0.033 | 0.699 |
|  | Monocytes | -0.064 | 0.699 |
|  | Neutrophils | -0.02 | 0.699 |
| cg25203704 | Basophils | -0.374 | 0.686 |
|  | Eosinophils | -0.041 | 0.686 |
|  | Lymphocytes | -0.032 | 0.686 |
|  | Monocytes | -0.062 | 0.686 |
|  | Neutrophils | -0.02 | 0.686 |
| cg16597045 | Basophils | -0.028 | 0.652 |
|  | Eosinophils | 0.002 | 0.814 |
|  | Lymphocytes | -0.003 | 0.49 |
|  | Monocytes | 0.002 | 0.814 |
|  | Neutrophils | -0.002 | 0.23 |
| cg23256822 | Basophils | 0.005 | 0.936 |
|  | Eosinophils | 0.025 | 0.195 |
|  | Lymphocytes | 0.004 | 0.477 |
|  | Monocytes | 0.007 | 0.725 |
|  | Neutrophils | 0.002 | 0.477 |
| cg03938978 | Basophils | -0.105 | 0.869 |
|  | Eosinophils | 0.092 | 0.495 |
|  | Lymphocytes | 0.016 | 0.869 |
|  | Monocytes | -0.01 | 0.869 |
|  | Neutrophils | 0.004 | 0.869 |
| cg12278959 | Basophils | -0.041 | 0.935 |
|  | Eosinophils | 0.046 | 0.355 |
|  | Lymphocytes | 0.007 | 0.935 |
|  | Monocytes | -0.002 | 0.935 |
|  | Neutrophils | <0.001 | 0.935 |
| cg27441011 | Basophils | -0.076 | 0.86 |
|  | Eosinophils | -0.003 | 0.892 |
|  | Lymphocytes | -0.004 | 0.86 |
|  | Monocytes | -0.009 | 0.86 |
|  | Neutrophils | -0.004 | 0.86 |
| cg02110603 | Basophils | -0.012 | 0.701 |
|  | Eosinophils | -0.002 | 0.701 |
|  | Lymphocytes | 0.005 | 0.09 |
|  | Monocytes | 0.009 | 0.337 |
|  | Neutrophils | 0.001 | 0.337 |

*CpG: cytosine-guanine; FDR: False Discovery Rate (0.05)*

**Table S3.** *Association between RNAs and left hippocampal tail in MDD and Control groups*

|  | MDD | | HC | |
| --- | --- | --- | --- | --- |
| RNA | Beta | p-value | Beta | p-value |
| BASP1-AS1 | -0.127 | 0.31 | 0.071 | 0.81 |
| MBOAT2 | -0.1 | 0.31 | <0.001 | 0.997 |
| ALPK1 | -0.092 | 0.31 | 0.056 | 0.81 |
| IL18RAP | -0.094 | 0.31 | -0.11 | 0.64 |
| LINCO1270 | 0.047 | 0.53 | -0.012 | 0.997 |
| C1orf200 | -0.089 | 0.31 | 0.117 | 0.64 |
| AGAP1 | 0.084 | 0.31 | 0.123 | 0.64 |

*HC: healthy control, MDD: Major Depressive Disorder*

**Table S4.** *Association between BASP1-AS1 expression and left hippocampal tail volumes in MDD*

| Predictor | Beta | p-value |
| --- | --- | --- |
| BASP-AS1 | -0.058 | 0.408 |
| Age | -0.033 | 0.680 |
| Total Brain Volume | 0.531 | < .001 |
| MADRS | -0.037 | 0.599 |
| Duration current MDE | -0.028 | 0.692 |
| Sex: |  |  |
| M – F | -0.18 | 0.360 |

*F: Female, M: Male, MADRS: Montgomery Asberg Depression Rating Scale, MDE: Major Depressive Episode*
